# Quantifying long-term stress in brown bears with the hair cortisol concentration: a biomarker that may be confounded by rapid changes in response to capture and handling

**DOI:** 10.1093/conphys/cou026

**Published:** 2014-07-16

**Authors:** Marc Cattet, Bryan J. Macbeth, David M. Janz, Andreas Zedrosser, Jon E. Swenson, Mathieu Dumond, Gordon B. Stenhouse

**Affiliations:** 1 Canadian Wildlife Health Cooperative, Western College of Veterinary Medicine, University of Saskatchewan, Saskatoon, Saskatchewan, Canada S7N 5B4; 2 Ecosystem and Public Health, Faculty of Veterinary Medicine, University of Calgary, Calgary, Alberta, Canada T2N 4Z6; 3 Department of Veterinary Biomedical Sciences, Western College of Veterinary Medicine, University of Saskatchewan, Saskatoon, Saskatchewan, Canada S7N 5B4; 4 Department of Environmental and Health Studies, Telemark University College, 3800 Bø, Norway; 5 Department of Ecology and Natural Resource Management, Norwegian University of Life Sciences, NO-1432 Ås, Norway; 6 Department of Environment, Government of Nunavut, Kugluktuk, Nunavut, Canada X0B 0E0; 7 Foothills Research Institute, Hinton, Alberta, Canada T7V 1X6

**Keywords:** Barbed-wire snag, body condition, capture, hair cortisol concentration, stress, *Ursus arctos*

## Abstract

The measurement of cortisol in hair is becoming important in studying the role of stress in the life history, health and ecology of wild mammals. The hair cortisol concentration (HCC) is generally believed to be a reliable indicator of long-term stress that can reflect frequent or prolonged activation of the hypothalamic–pituitary–adrenal axis over weeks to months through passive diffusion from the blood supply to the follicular cells that produce the hair. Diffusion of cortisol from tissues surrounding the follicle and glandular secretions (sebum and sweat) that coat the growing hair may also affect the HCC, but the extent of these effects is thought to be minimal. In this study, we report on a range of factors that are associated with, and possibly influence, cortisol concentrations in the hair of free-ranging brown bears (*Ursus arctos*). Through two levels of analyses that differed in sample sizes and availability of predictor variables, we identified the presence or absence of capture, restraint and handling, as well as different methods of capture, as significant factors that appeared to influence HCC in a time frame that was too short (minutes to hours) to be explained by passive diffusion from the blood supply alone. Furthermore, our results suggest that HCC was altered after hair growth had ceased and blood supply to the hair follicle was terminated. However, we also confirmed that HCC was inversely associated with brown bear body condition and was, therefore, responsive to diminished food availability/quality and possibly other long-term stressors that affect body condition. Collectively, our findings emphasize the importance of further elucidating the mechanisms of cortisol accumulation in hair and the influence of long- and short-term stressors on these mechanisms.

## Abstract (precis)

We evaluated the effects of several factors on the cortisol concentration of hair in wild brown bears. Our findings suggest the cortisol concentration may increase quickly following capture, even after hair growth has ceased. We emphasize the need for more validation of this measure as an indicator of long-term stress.

## Introduction

Stress is increasingly recognized as an influential factor in the life history, health and ecology of wild animals. Most research concerns how the hypothalamic–pituitary–adrenal axis, hereinafter called the central stress axis, a major pathway of the neuroendocrine system, is affected by ecological factors (Clinchy *et al.*, 2013; Wingfield, 2013), including those related to human activities (Wasser *et al.*, 2011; Wingfield *et al.*, 2011), and how it affects life history (Dantzer *et al.*, 2013; Sheriff and Love, 2013), individual health and fitness (Acevedo-Whitehouse and Duffus, 2009; Busch and Hayward, 2009; Schultner *et al.*, 2013) and population performance (Jachowski *et al.*, 2012; Strasser and Heath, 2013). Stress is usually quantified by measuring aspects of the stress response, which are either elements of the central stress axis (e.g. glucocorticoids) or effects of central stress axis activation (e.g. immune cell trafficking, stress protein synthesis), or a combination of the two. However, a persistent challenge in interpreting the magnitude of the stress response is distinguishing long-term (or chronic) changes that occur over weeks, months or longer because of increased production of cortisol caused by environmental stressors (e.g. loss of habitat and food, increased human disturbance) from normal daily and seasonal modulations in cortisol production, including natural adjustments associated with reproduction (Boonstra, 2004). Other research has measured physiological processes that are negatively affected by stress. These include studies of oxidative damage (Monaghan *et al.*, 2009; Selman *et al.*, 2012), telomere dynamics (Barrett *et al.*, 2013; Mizutani *et al.*, 2013), fluctuating asymmetry (Allenbach, 2011; Sánchez-Chardi *et al.*, 2013) and changes in body mass or body condition (Hodges *et al.*, 2006; Sheriff *et al.*, 2011b). All these studies, irrespective of whether stress is considered as a response or an effect, require robust indices of stress that are sensitive to changes in stressor frequency and intensity, and that can be interpreted in the face of confounding factors, e.g. age, sex, season. This means that these indices should be fully validated to ensure an acceptable degree of accuracy and precision in their application and interpretation (Sheriff *et al.*, 2011a; Johnstone *et al.*, 2012).

Measurement of the glucocorticoids cortisol and corticosterone in hair and feathers, respectively, has been recognized as a potentially important advancement in studying the role of stress in wild animals (Koren *et al.*, 2002; Bortolotti *et al.*, 2008; Sheriff *et al.*, 2011a). A recent increase in the number of peer-reviewed articles reporting on the application of these measurements in free-ranging wildlife seems to support this view; 22 articles have been published over the past 3 years compared with 10 articles in the preceding 9 years, starting with the first report by Koren *et al.* in 2002 (Wildlife and Ecology Studies Worldwide Database, 2013 EBSCO Industries, Inc., Ipswich, MA, USA). The reasons for this broadening application are 2-fold. The use of hair and feathers as biological samples offers several advantages when compared with the use of other tissues. Both hair and feathers can be collected from animals without capturing them, e.g. with the use of hair traps (Woods *et al.*, 1999) or the gathering of moulted feathers from breeding and nesting sites (Miño and Del Lama, 2009). This allows researchers to circumvent the potentially confounding influence of stress caused by capture and handling, it reduces the likelihood of adversely affecting the welfare and fitness of study animals, and it enables cost-effective collection from large numbers of animals over large areas. Hair and feather samples are also easy and inexpensive to prepare for storage; they are simply air-dried, sealed in paper envelopes and stored at indoor ambient temperature (Bortolotti *et al.*, 2008; Macbeth *et al.*, 2010). Samples also can be assigned to individual animals through DNA extraction and analysis (Miño and Del Lama, 2009; Proctor *et al.*, 2010). Finally, because hair cortisol and feather corticosterone do not degrade appreciably over time or after exposure to the environment (Bortolotti *et al.*, 2009; Macbeth *et al.*, 2010), this opens the possibility of conducting analyses using archived samples, including museum specimens. The other reason why measurement of glucocorticoids in hair and feathers is being applied increasingly is that the amounts of glucocorticoid in these matrices are thought to chronicle frequent or prolonged central stress axis activation that is over and above the stress axis activity associated with normal biological, daily and seasonal events, on a time scale of weeks to months. This contrasts with the time scale of hours or days reflected by other matrices, including blood (serum or plasma), urine, saliva and faeces. Of these, faeces have also been widely used for the ‘non-invasive’ measurement of stress as reflected by its glucocorticoid levels (Wasser *et al.*, 2000, 2011; Rodrigues da Paz *et al.*, 2014). However, use of faecal glucocorticoid concentrations as an index of stress in brown bears has proved to be particularly challenging due to complex seasonal and dietary influences (von der Ohe *et al.*, 2004). Overall, the collection of hair or feathers and measurement of the cortisol/corticosterone concentration appears to be particularly well suited for the investigatiion of long-term (or chronic) stress (Sheriff *et al.*, 2011a), and this type of stress is often of more interest than short-term (or acute) stress in studies examining how wild animals fare with environmental change, over-exploitation and biological invasion (e.g. Anson *et al.*, 2013; Bechshøft *et al.*, 2013; Zwijacz-Kozica *et al.*, 2013).

However, hair cortisol and feather corticosterone have not been fully validated as indicators of long-term stress in wildlife. Although glucocorticoids can be measured reliably in these matrices, and their concentrations following moult or collection by barbed wire remain stable with time and are resistant to weather-induced change (Bortolotti *et al.*, 2008, 2009; Macbeth *et al.*, 2010), the exact mechanism(s) of glucocorticoid integration are undetermined. A major route of integration has been hypothesized to be through passive diffusion from the vascular supply to the follicular cells that produce the hair or feather shaft (Sharpley *et al.*, 2011; Russell *et al.*, 2012). In fact, this is sometimes presumed to be the only route of integration in the wildlife literature (e.g. Bortolotti *et al.*, 2008; Koren *et al.*, 2008; Fourie and Bernstein, 2011; Terwissen *et al.*, 2013). The consequences arising from this assumption are as follows: (i) cortisol integration is determined by the dynamics of unbound (free) cortisol in the bloodstream combined with the rate of hair/feather growth; (ii) cortisol integration ceases when the hair/feather stops growing; (iii) cortisol concentrations in hair/feather reflect an integrated record of central stress axis activation during the period of hair/feather growth; and (iv) cortisol concentrations in hair/feather will not be affected by the stress of capture, restraint and handling provided that hair/feather samples are collected concurrently, thus preventing further growth and integration. However, several recent studies of non-wildlife species, including humans, suggest that cortisol in hair may be derived from the skin, in addition to that drawn from the systemic circulation (Sharpley *et al.*, 2010a; Keckeis *et al.*, 2012). The source of this cortisol production is believed to be a parallel, but peripheral (in contrast to central), stress axis within skin, including its epidermal and dermal compartments, as well as hair follicles (Slominski and Mihm, 1996; Ito *et al.*, 2005; Slominski *et al.*, 2007). The existence of a similar peripheral stress axis within the skin of birds has been hypothesized (e.g. Koren *et al.*, 2012; Lendvai *et al.*, 2013), but not confirmed. It should also be noted that cortisol arising from either or both axes may also be deposited onto the outside of the hair shaft in association with sebum and sweat, but until recently it was not known whether this ‘external’ cortisol could be incorporated into the shaft (Meyer and Novak, 2012; Stalder and Kirschbaum, 2012). Recently, however, Russell *et al.* (2014) demonstrated that profuse sweating in human subjects after intense exercise may increase the cortisol concentrations detected in hair.

The implications of the presence of a peripheral stress axis in mammals are uncertain. If it is fully synchronous with the central stress axis and passive diffusion is the only route of cortisol incorporation into the hair, then the consequences, as stated above, should remain unchanged. However, the consequences become less certain if the two stress axes are only partly synchronous or completely asynchronous, if cortisol incorporation also involves active transport, and/or if cortisol from sebum and sweat is readily integrated into the hair shaft. While the existence of a functional peripheral stress axis is widely accepted, viewpoints still vary on what is the major determinant of the cortisol concentration in hair. Some believe that, although the peripheral stress axis may contribute marginally, the central stress axis is the primary contributor (Russell *et al.*, 2012; Stalder and Kirschbaum, 2012). The contrary opinion is that the cortisol concentration in hair may be independently influenced at some times by the peripheral stress axis, concurrently influenced by both axes at other times, and that the linkages between, and co-ordination of, the two axes are not yet fully understood (Sharpley *et al.*, 2011; Zmijewski and Slominski, 2011). This latter view recognizes the possibility that cortisol concentrations along the hair shaft may represent a dynamic process, in which cortisol integration changes immediately and frequently in response to environmental threats of long or short duration (Thomson *et al.*, 2009; Sharpley *et al.*, 2010b).

Herein, we report on a range of factors that are associated with, and possibly influence, cortisol concentrations in the hair of free-ranging brown bears (*Ursus arctos*). Some of our findings challenge the assumption that passive diffusion from the vascular supply to the follicular cells that produce the hair is the only significant route of integration. At the very least, these results should serve to emphasize the need for further validation studies to determine whether the hair cortisol concentration (HCC) can be applied with confidence as a long-term stress indicator in wildlife investigations.

## Materials and methods

### Sources of brown bear hair

**Figure 1:**
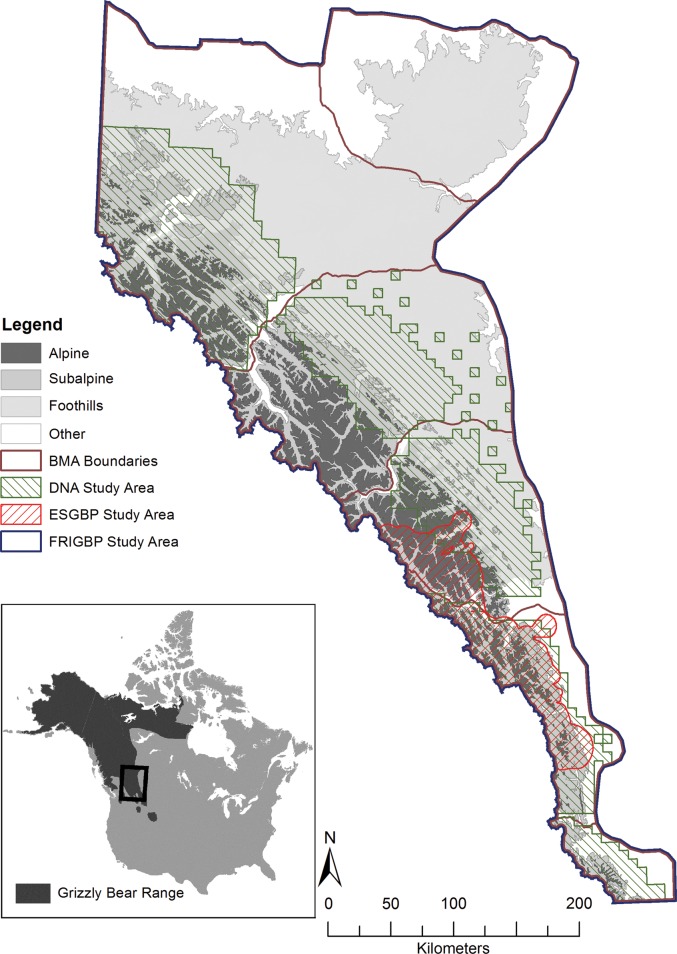
Brown bear study areas in Alberta, Canada from 1994 to 2012. Hair samples were collected following the capture of 53 unique animals in the Eastern Slopes Grizzly Bear Project (ESGBP) study area (from 1994 to 2002) and from 125 unique animals in the Foothills Research Institute Grizzly Bear Project (FRIGBP) study area (from 2001 to 2012). Hair samples also were collected by barbed-wire snagging from 323 unique animals in the Alberta Grizzly Bear DNA Inventory study area from 2004 to 2008. Abbreviation: BMA, bear management area. Map projection, Mercator.

We obtained 505 hair samples collected from three independent projects studying brown bears throughout their distributional range in Alberta, Canada, from 1994 to 2012 (Fig. 1). These represented 486 unique individual bears, from which 19 individuals were sampled on two occasions. Although hair collection methods varied slightly by project (details below), hair samples were handled in a similar manner in that they never made contact with human skin. They were either placed into a paper envelope using forceps or by hand when wearing sterile examination gloves. The envelopes were left open for several hours to ensure that the samples were air-dried, and then sealed and stored under low light at room temperature (∼20°C) until the time of laboratory analysis. Details of each project are as follows.

#### Eastern Slopes Grizzly Bear Project (ESGBP)

This study was conducted from 1994 to 2002 with the goal of contributing towards a scientific understanding of brown bear biology, ecology and demography in an area of ∼40 000 km^2^ in west-central Alberta (Fig. 1) and east-central British Columbia known as the Central Canadian Rocky Mountain Ecosystem (50–52°N, 114–117°W; Herrero, 2005). Hair samples were collected from live-captured bears, with details on capture and handling procedures provided by Garshelis *et al.* (2005). All hair samples were plucked from the top of the shoulders, in the area of the prominent shoulder hump, using a haemostatic clamp. In addition to obtaining hair samples from 53 bears that were captured in Alberta, we were also provided with data regarding their identity, sex, age, reproductive status of females (accompanied by offspring or not), location, body mass, body length and method of capture (culvert trap or leg-hold snare). We estimated body condition for bears using a body condition index (BCI), for which mass is standardized relative to length (Cattet *et al.*, 2002).

#### Foothills Research Institute Grizzly Bear Program (FRIGBP)

This study, now in its 16th year, was initiated in 1999 to provide knowledge and planning tools to land and resource managers to ensure the long-term conservation of brown bears in Alberta (Stenhouse and Graham, 2011). The study area of ∼300 000 km^2^ encompasses the entire distributional range of brown bears within the province (49–58°N, 113–120°W), with yearly research effort typically targeted toward one or two bear management areas (BMAs; Fig. 1). For the present study, we selected the HCC data from 125 bears captured from 2001 to 2012, with details on capture and handling procedures provided by Cattet *et al.* (2008). All hair samples were collected from the top of the shoulders by use of a haemostatic clamp to pull hairs from the skin, or by use of either bandage scissors or electric clippers to cut hair at the skin. We also had the identity, sex, age, location of capture, body mass, body length, BCI and capture method (remote drug delivery from helicopter, culvert trap or leg-hold snare) for each bear. In addition, we had restraint times (time elapsed from capture to initiation of anaesthesia) for 22 bears captured by either culvert trap or leg-hold snare based on the use of trap-timing devices (Blue Oceans Satellite Systems Inc., St John's, Newfoundland, Canada) that were activated when a bear caused a trap to spring.

#### Alberta Grizzly Bear DNA Inventory

As part of brown bear management and recovery in Alberta (Canada), the provincial government and federal jurisdictional partners conducted DNA inventories from 2004 to 2008 to estimate population size and density for five BMAs (Fig. 1; Alberta SRD, 2010). The total area covered ∼132 000 km^2^, mostly in and adjacent to the eastern slopes of the Rocky Mountains, from northern Montana (49°N, 113°W) to the city of Grande Prairie (55°N, 118°W). Hair was collected by barbed-wire snagging during June and July of each year, with study design details provided in a series of technical reports (Boulanger *et al.*, 2005a, 2005b, 2007, 2008). In 2004, a perimeter barbed-wire fence was set surrounding a bait site, with two parallel strands set at 50 and 75 cm above the ground. In subsequent years, barbed wire was limited to a single strand set at 60 cm above the ground. Locations of sample collection sites were determined by Global Positioning System (GPS) receiver, and suitable samples were genotyped to confirm species, sex and individual identity (Paetkau, 2003). Hair samples from 308 unique individuals were used for the determination of cortisol concentration.

### Laboratory analysis of hair cortisol concentration

We used only guard hairs with the follicles removed to determine HCC, as recommended by Macbeth *et al.* (2010). Surface contamination was removed by washing hairs with methanol (three 3 min washes), as described in detail elsewhere (Macbeth *et al.*, 2010). Following decontamination, hair was dried, ground to a fine powder using a ball mill, and weighed. Ground hair samples were immersed in 0.5 ml of high-resolution gas chromatography-grade methanol, gently swirled (10 s), and placed on a slowly spinning rotator to extract for 24 h. Following extraction, samples were centrifuged for 15 min at 2150 *g*, the methanol extract was removed, evaporated until dryness under nitrogen gas (38°C), and reconstituted in phosphate buffer (0.2 ml). Cortisol was quantified as picograms of cortisol per milligram of washed and dried hair (pg/mg) using a commercially available enzyme-linked immunoassay kit (Oxford EA-65 Cortisol EIA kit; Oxford Biomedical, Lansing, MI, USA), which was previously validated for use in brown bears (Macbeth *et al.*, 2010).

### Statistical analysis

We used generalized additive mixed models (GAMMs; Wood, 2006; Zuur *et al.* 2014) in two separate analyses, a coarse-level and a fine-level analysis, to evaluate relationships between the natural logarithmically (ln) transformed response variable, HCC, and a range of potential predictor variables (Table 1). In the coarse-level analysis, we used the maximal number of HCC values available for unique individuals (*n* = 486), but the number of potential predictor variables was limited to sex, location (BMA), year of collection and method of collection, because many of the samples had been collected by barbed-wire snagging (*n* = 308), hence no information was available concerning age or physical attributes. In the fine-level analysis, we used a smaller number of HCC values (*n* = 116) with the widest number of potential predictor variables possible by excluding HCC values from samples collected by barbed-wire snagging as well as values from bears that were captured but lacked a full complement of supplementary data (Table 1). In both analyses, the identity of each bear was included as a random effect and, for bears captured more than once, we used only data collected at the last capture, i.e. no repeated measures.

**Table 1:** The attributes and variables evaluated as potential determinants of the cortisol concentration in hair samples collected from brown bears in Alberta, Canada from 1994 to 2012

Attribute	Variable	Variable description	Coarse-level analysis (*n* = 486)	Fine-level analysis (*n* = 116)
Biology	Sex	*Factor* (female, male)	Yes	Yes
	Dependent offspring	*Factor* (female only: yes or no)	No	Yes
	Age	*Covariate* (2–22 years)	No	Yes
Time	Year	*Covariate* (1994–2012)	Yes	Yes
	Julian day	*Covariate* (107–293)	No	Yes
Location	Bear management area (BMA)	*Factor* (Grande Cache, Yellowhead, Clearwater, Livingstone, Castle)	Yes	Yes
Growth	Body mass	*Covariate* (45–311 kg)	No	Yes
	Contour length	*Covariate* (137–222 cm)	No	Yes
	Body condition	*Covariate* (body condition index: −2.12 to +2.86)	No	Yes
Collection	Hair collection method	*Factor* (barbed-wire hair snag with no capture, shaved following capture by remote drug delivery from helicopter, shaved following capture by culvert trap, shaved following capture by leg-hold snare)	Yes	No
	Capture method	*Factor* (remote drug delivery from helicopter, culvert trap, leg-hold snare)	No	Yes

Prior to conducting the analyses, we carried out the following preliminary assessment: (i) we constructed Cleveland dot plots to evaluate the response variable and covariates for outliers; and (ii) we measured the Pearson correlation (*r*) between, and the variance inflation factors (VIFs) for, predictor covariates to prevent multicollinearity, i.e. *r* < 0.7 and VIF < 3. Following this, we centred continuous covariates to aid interpretation of parameter coefficients (Schielzeth, 2010).

For both analyses, we followed the approach described by Zuur (2012) to select the most parsimonious model of all possible combinations of the variables and their interactions, including a null model, based on differences in the Akaike's information criteria corrected for small sample sizes (ΔAIC_c_; Anderson, 2008). We used the ‘mgcv’ package (Wood, 2013) in R 3.0.1 (R Development Core Team, 2013) for statistical analyses. We validated the most parsimonious models by evaluating the distribution of standardized model residuals for normality, and by plotting standardized model residuals vs. the covariate values to ensure that the residuals were scattered at random around the horizontal line at zero (Zuur *et al.*, 2009).

We used a mixed-model analysis of repeated measures (‘mixed model analysis’ procedure in IBM^®^ SPSS^®^ Statistics Version 20; IBM North America, New York, NY, USA) to compare ln-transformed HCC values for 19 bears that were sampled two or three times, once by barbed wire and once or twice following capture, with the sequence of sampling method and years of sampling varying among bears. Both collection method (hair snag and capture) and year (2003–12) were modelled as fixed effects, whereas bear identity was modelled as a random effect. To avoid repeated measures, only HCC values from the last sampling event for these bears were used in the coarse-level and/or fine-level analyses.

We calculated the Pearson correlation coefficient to evaluate the linear association between ln-transformed restraint time and HCC for 17 bears captured by culvert trap. We also evaluated a scatterplot of these data for evidence of any non-linear association.

## Results

**Figure 2:**
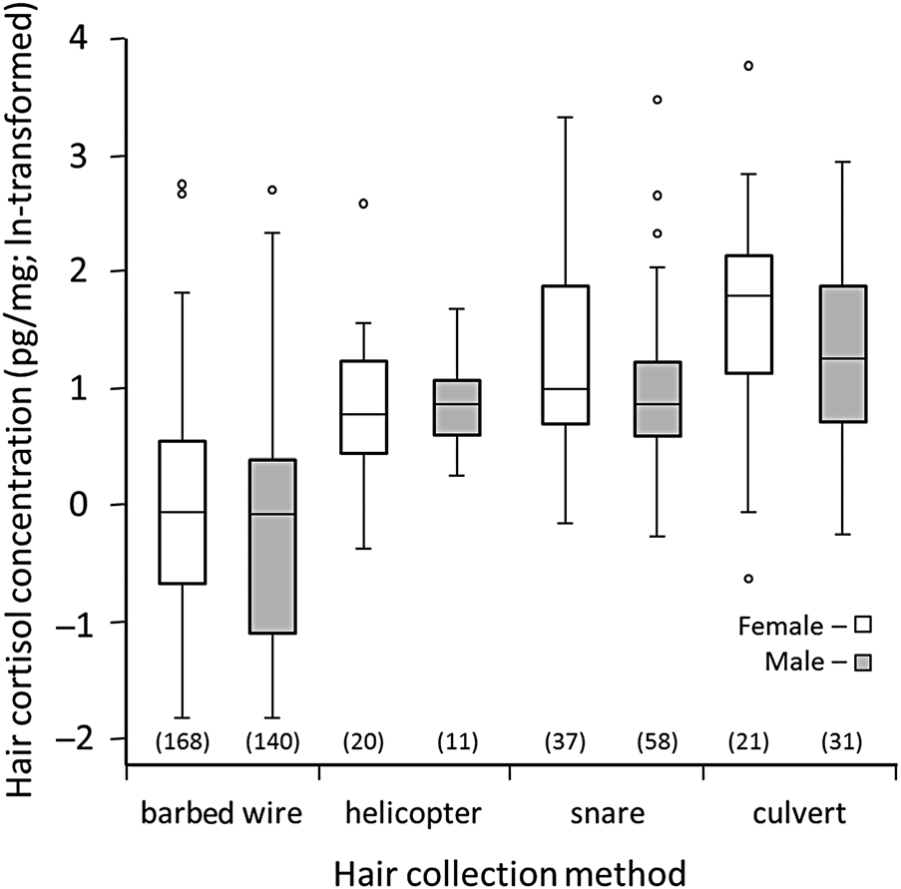
Box plot comparison of hair cortisol concentration (HCC) among female and male brown bears (*n* = 486) sampled by barbed-wire hair snagging (barbed wire) or by clipping hair following capture by remote drug delivery from helicopter (helicopter), leg-hold snare (snare) or culvert trap (culvert). The top and bottom of each box represents the first and third quartiles (Q1 and Q3), while the inside line represents the median. The vertical line goes to the first data points before the ‘1.5’ cut-off above and below the box. The ‘1.5’ cut-off above is calculated as Q3 + 1.5 × (Q3 − Q1) and the ‘1.5’ cut-off below is calculated as Q1 − 1.5 × (Q3 − Q1). The points represent values (outliers) lying outside of the range defined by the vertical lines. Sample sizes for sex × hair collection method categories are presented in parentheses below each box plot.

In the coarse-level analysis, we found the strongest support for a model that included sex, year of hair collection and method of hair collection as predictor variables (Table 2). Male bears tended to have lower HCC than females (Table 3 and Fig. 2). Hair cortisol concentration varied among years, but without any clear temporal pattern. Cortisol concentrations were significantly lower in hair samples collected by barbed wire than in hair samples collected immediately following capture (Table 3 and Fig. 2). Among hair samples collected following capture, cortisol concentrations were highest in bears captured by culvert trap, but similar between bears captured by remote drug delivery from helicopter and bears captured by leg-hold snare (Table 3 and Fig. 2). We also found support (ΔAIC_c_ ≤ 2.00) for a model that included the method of hair collection and year, but excluded sex (Table 2). Support was lacking for a global model that included all variables (ΔAIC_c_ = 6.09) and a null model that included only the intercept (ΔAIC_c_ = 207.22). There also was less support (ΔAIC_c_ = 4.75) for a generalized linear form of the strongest model (Model 1 in Table 2).

**Table 2:** Model selection results for the coarse-level analysis (*n* = 486) of factors affecting hair cortisol concentration of brown bears in Alberta, Canada

Model (*i*)	Candidate models	*k*	AIC_c_	ΔAIC_c_	*w*_*i*_	*R*^2^
**1**	**Sex + year + collection**	**4**	**1313**.**53**	**0**.**00**	**0**.**59**	**0**.**36**
**2**	**Year + collection**	**3**	**1315**.**51**	**1**.**98**	**0**.**22**	**0**.**35**
3	Year + day + collection	4	1317.03	3.50	0.10	–
4	Sex + year + BMA + collection	5	1318.46	4.93	0.05	–
5	Global model (all variables)	6	1319.62	6.09	0.03	–
6	Sex + collection	3	1323.38	9.85	0.00	–
7	BMA + collection	3	1323.66	10.13	0.00	–
8	Year + day + sex	4	1346.57	33.04	0.00	–
9	Year + day	3	1347.83	34.30	0.00	–
10	Year + day + BMA	4	1348.30	34.78	0.00	–
11	Null model (intercept only)	1	1520.74	207.22	0.00	–

Sample-size-adjusted Akaike information criteria (AIC_c_), number of parameters (*k*), difference in AIC_c_ between most supported and given model (ΔAIC_c_), Akaike weight of evidence supporting the *i*th model (*w*_*i*_), and the adjusted coefficient of determination for models where ΔAIC_c_ ≤ 2.00 (*R*^2^). Bold numbers denote supported models where ΔAIC_c_ ≤ 2.00. Abbreviation: BMA, bear management area.

**Table 3:** Parameter estimates for the top model predicting hair cortisol concentration from Table 2

Parameter	β_*i*_	SE	e.d.f.	*P* value
Intercept	1.06	0.170	–	** ≤ 0**.**001**
Sex (male vs. female)	−0.17	0.085	–	**0**.**049**
Collection (barbed-wire hair snag vs. helicopter capture)	−1.23	0.184	–	** ≤ 0**.**001**
Collection (culvert trap vs. helicopter capture)	0.66	0.208	–	** ≤ 0**.**001**
Collection (leg-hold snare vs. helicopter capture)	0.04	0.200	–	0.827
s(year)	–	–	4.34	**0**.**011**

Parameter coefficient (β_*i*_), standard error (SE), estimated degrees of freedom (e.d.f.) for the spline function (s), and the statistical significance (*P*). Bold numbers denote significant parameters where *P* ≤ 0.05.

To correct for incongruity between the timing of the Alberta Grizzly Bear DNA Inventory (2004–08) and the two research projects (1994–2012), we re-ran the coarse-level analysis for Model 1 in Table 2 using data restricted to the years 2004–08. We also combined the data for capture by remote drug delivery from helicopter and capture by leg-hold snare into a single category to strengthen the comparison of mean HCC between bears sampled following capture and free-ranging bears that were snagged by barbed wire. Again, the findings were similar (GAMM results not shown). Bears sampled following capture either by remote drug delivery from helicopter or by leg-hold snare were more likely to have a greater HCC (non-transformed median: 2.38 pg/mg; *n* = 53, β = 1.17 ± 0.147, *P* ≤ 0.001) than that for free-ranging bears sampled by barbed wire (0.94 pg/mg; *n* = 303). Bears sampled following capture by culvert trap were more likely to have a higher HCC (6.58 pg/mg; *n* = 12, β = 1.97 ± 0.288, *P* ≤ 0.001) than that for free-ranging bears sampled by barbed wire (0.94 pg/mg; *n* = 303) and were also more likely to have a higher HCC (6.58 pg/mg; *n* = 12, β = 0.80 ± 0.313, *P* = 0.011) than that for bears sampled following capture either by remote drug delivery from helicopter or by leg-hold snare (2.38 pg/mg; *n* = 53).

We also re-ran the coarse-level analysis for Model 1 in Table 2 using HCC data from bears captured prior to 16 May (Julian day <137) and following 15 October (Julian day >288) to evaluate HCC levels during the time of year when the hair growth cycle is believed to be in a resting phase (Hilderbrand *et al.*, 1996; Jacoby *et al.*, 1999). Again, bears sampled following capture by any of the three methods were more likely to have a higher HCC (median = 2.30 pg/mg; *n* = 47, β = 1.12 ± 0.170, *P* ≤ 0.001) than that for free-ranging bears sampled by barbed wire (0.94 pg/mg; *n* = 303).

**Figure 3:**
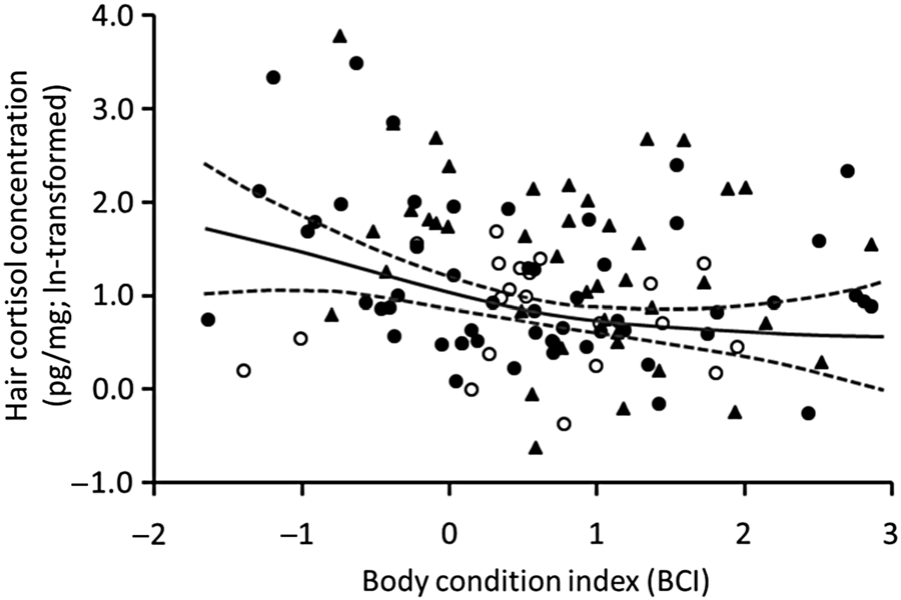
The association between hair cortisol concentration and body condition index (BCI) values for 116 brown bears captured in Alberta, Canada. The continuous curved line is the estimated smoother for the BCI taken from the additive mixed model in Table 5, the dashed curved lines are point-wise 95% confidence bands, and the points are the observed values for bears captured by remote drug delivery from helicopter (open circles), leg-hold snare (filled circles) or culvert trap (filled triangles).

In the fine-level analysis, we found strongest support for a model that included age, body condition, year of capture, Julian day of capture and method of capture as predictor variables (Table 4). Age ranged from 2 to 22 years, with wide variation in HCC across most ages (Table 5). Hair cortisol concentration was generally higher in bears that were in poorer body condition, i.e. BCI ≤ 0.6 (Table 5 and Fig. 3). This association did not appear to be confounded by capture, because the range and distribution of BCI values were similar within each method of capture (Fig. 3). As in the coarse-level analysis, HCC varied significantly among years, but without any obvious pattern. The HCC values also tended to be higher in bears captured from July to October (*n* = 12) than in bears captured in June or earlier (*n* = 104). Differences in HCC among methods of capture remained similar to our findings in the coarse-level analysis, with HCC values often being higher in bears captured by culvert trap, but similar between bears captured by remote drug delivery from helicopter and bears captured by leg-hold snare (Table 5 and Fig. 3). We also found support (ΔAIC_c_ ≤ 2.00) for two other models (Models 2 and 3) that, like Model 1, included body condition, year of capture, Julian day of capture and method of capture as predictor variables (Table 4). Other predictor variables included in Model 3 were not significant (age, *P* = 0.242; and body length, *P* = 0.697). We did not find support for any other models, including a global model (ΔAIC_c_ = 9.32), a null model (ΔAIC_c_ = 23.49) or models with interaction terms (Models 6 and 8). We also did not find support (ΔAIC_c_ = 10.77) for a generalized linear form of the strongest model (Model 1 in Table 4).

**Table 4:** Model selection results for the fine-level analysis (*n* = 116) of factors affecting hair cortisol concentration of brown bears in Alberta, Canada

Model (*i*)	Candidate models	*k*	AIC_c_	ΔAIC_c_	*w*_*i*_	*R*^2^
**1**	**Age + body condition + year + day + capture**	**6**	**269**.**37**	**0**.**00**	**0**.**44**	**0**.**25**
**2**	**Body condition + year + day + capture**	**5**	**270**.**02**	**0**.**65**	**0**.**32**	**0**.**24**
**3**	**Age + body condition + length + year + day + capture**	**7**	**270**.**97**	**1**.**60**	**0**.**20**	**0**.**25**
4	Year + day + capture	4	275.66	6.29	0.02	–
5	Age + body condition + length + year + day + BMA + capture	8	276.44	7.08	0.01	–
6	Sex + age + body condition + (sex × body condition) + year + day + capture	10	278.55	9.19	0.00	–
7	Global model (all variables)	9	278.69	9.32	0.00	–
8	Sex + age + body condition + (sex × age) + year + day + capture	10	279.35	9.98	0.00	–
9	Year + day	3	282.59	13.22	0.00	–
10	Body condition + length	3	287.03	17.66	0.00	–
11	Capture	2	289.08	19.72	0.00	–
12	Null model (intercept only)	1	292.86	23.49	0.00	–

Sample-size-adjusted Akaike information criteria (AIC_c_), number of parameters (*k*), difference in AIC_c_ between most supported and given model (ΔAIC_c_), Akaike weight of evidence supporting the *i*th model (*w*_*i*_) and the adjusted coefficient of determination for models where ΔAIC_c_ ≤ 2.00 (*R*^2^). Bold numbers denote supported models where ΔAIC_c_ ≤ 2.00. Abbreviation: BMA, bear management area.

**Table 5:** Parameter estimates for the top model predicting hair cortisol concentration from Table 4

Parameter	β_*i*_	SE	e.d.f.	*P* value
Intercept	0.81	0.175	–	** ≤ 0**.**001**
Capture (culvert trap vs. helicopter capture)	0.76	0.212	–	** ≤ 0**.**001**
Capture (leg-hold snare vs. helicopter capture)	0.17	0.232	–	0.472
s(age)	–	–	1.72	0.231
s(body condition)	–	–	1.87	**0**.**025**
s(day)	–	–	1.00	0.074
s(year)	–	–	3.32	**0**.**007**

Parameter coefficient (β_*i*_), standard error (SE), estimated degrees of freedom (e.d.f.) for the spline function (s), and the statistical significance (*P*). Bold numbers denote significant parameters where *P* ≤ 0.05.

Nineteen bears were sampled two or three times, once by barbed-wire snagging and once or twice following capture by one of the three methods (remote drug delivery by helicopter, leg-hold snare or culvert trap). The HCC values were significantly higher (mixed-model analysis of repeated measures: *F*_1,17.5_ = 57.78, *P* ≤ 0.001) in samples collected following capture (mean ± SEM: 3.14 ± 0.29 pg/mg, *n* = 23) than in samples collected by barbed wire (2.31 ± 1.20 pg/mg, *n* = 19), whereas year of sampling was not significant (*F*_8,17.2_ = 1.37, *P* = 0.279). The time between collections of hair samples by barbed-wire snag and by capture from 10 bears was >1 year and, therefore, represented different hair cycles. The time between collections from six bears was between 11 and 12 months, so we could not be certain whether samples represented the same or sequential hair cycles. Although a limited sample size, there were three paired samples that were collected <1 day apart, first by barbed-wire snagging and then following capture by culvert trap, and therefore in the same hair cycle. The HCC for these three paired samples also was higher following capture than following barbed-wire snagging (bear one, 4.71 vs. 2.51 pg/mg; bear two, 3.01 vs. 2.64 pg/mg; and bear three, 2.03 vs. 1.66 pg/mg).

Restraint times for 17 bears captured by culvert trap ranged from 0.95 to 14.25 h, with an average time of 6.40 ± 1.30 h. We found no evidence of a linear association between ln-transformed values for restraint time and HCC (*r* = 0.024, *P* = 0.928, *n* = 17), and an evaluation of the scatterplot did not reveal a non-linear pattern in the data.

## Discussion

Our most important finding was that cortisol concentrations in hair were higher in bears following capture than in free-ranging bears that were snagged by barbed wire. This has not been reported previously, and it challenges the assumption that HCC is determined primarily by passive diffusion from the vascular supply to the follicular cells that produce the hair in two ways. First, HCC appeared to be affected by capture-induced stress too rapidly to be explained by cortisol integration with hair growth. Second, because some of the captures in this study occurred when the hair growth cycle was in a quiescent (telogen) phase, HCC also appeared to be affected by capture-induced stress after hair growth had terminated and blood flow to follicles had ceased.

Although we lack direct evidence to show that HCC increased quickly in response to capture, restraint and handling, our findings in the present study were unlikely to be caused by temporal or spatial effects. From a temporal standpoint, even when we constrained the coarse-level analysis to data collected only during the years of the Alberta Grizzly Bear DNA Inventory, 2004–08, the median HCC for bears captured by remote drug delivery from helicopter or by leg-hold snare was 2.5 times greater than that for free-ranging bears sampled by barbed wire, whereas the median HCC for bears captured by culvert trap was seven times greater. From a spatial standpoint, the three study areas overlapped on a broad regional scale (Fig. 1), but it is conceivable that captured bears occupied different types of habitats than free-ranging bears on a more local scale. Nevertheless, the data from 19 bears that were both snagged by barbed wire and sampled immediately following capture showed that HCC values were significantly higher following capture than when snagged, while values were not influenced appreciably by the year of hair collection. We also had data from an additional 22 bears that were both snagged and captured, but lacked HCC values following capture. Thus, in total, 41 of 308 bears (13%) were sampled following capture as well as by barbed wire, which suggests that capture and inventory procedures also overlapped at the level of home ranges.

Systematic differences in the collection and processing of hair samples from captured bears and hair samples from snagged bears also seems an unlikely explanation for the findings in the present study. Hair cortisol concentration does vary in individual animals depending on where the hair is collected from on the body (Macbeth *et al.*, 2010). Thus, a systematic bias is possible if the site of hair sampling differed consistently between the two methods of collection. Indeed, there probably was a difference in sampling site between methods in that samples collected from captured bears were consistently taken from the dorsum (upper side of the body), mostly in the area of the shoulder hump. In contrast, bears encountering barbed wire were snagged from varying body locations depending on whether they stepped over the barbed-wire strand, in which case hair was snagged from the ventrum (under side of the body) anywhere from the chest to the inside of the thighs, or if they crawled under the barbed-wire strand, in which case hair was snagged anywhere from the top of the neck to the rump. Macbeth *et al.* (2010) evaluated HCC levels in 15 brown bears that were sampled from four body regions (the neck, shoulders, rump and abdomen) and found that the only region to show consistent differences was the top of the neck, mid-way between the shoulders and head, where the HCC was ∼1.3–1.8 pg/mg higher than that measured in other regions. Given that samples from captured bears were generally collected from the top of the shoulders, but possibly the neck on some occasions, and given that barbed-wire-sourced samples were snagged from various body locations including the neck, it seems unlikely that a systematic sampling site bias could explain the differences in HCC values between captured bears and snagged bears. Nonetheless, we are now routinely collecting hair samples from three sites (top of the neck, shoulder and chest) on captured bears to provide a better understanding of the magnitude and consistency of differences between body regions.

Regarding potential systematic biases in the processing of samples, we analysed samples for the present study intermittently over a period of 5 years in batches of 10–50 samples per session. Thus, the samples were not analysed in a random manner. Nevertheless, we are confident there was no systematic interassay bias in HCC results on the basis that batches were often mixed, containing samples from both captured and snagged bears. In addition, we measured variation among samples run on different enzyme immunoassay plates on different days at the beginning of the study, and found that the coefficient of variation (%CV) was 5.75% (Macbeth *et al.*, 2010), suggesting that the day-to-day precision in the technique used to quantify HCC was high, i.e. %CV < 15.5% (Harris, 1988).

The finding that HCC values differed among capture methods also points towards the possibility that HCC may increase quickly in response to capture-related stress. This is based on the assumption that some methods of capture induce more or less stress than others, an assumption that has been verified from previous reports documenting differences between the physiological response of brown bears to capture by remote drug delivery from helicopter vs. their response to capture by leg-hold snare (Cattet *et al.*, 2003; Chow *et al.*, 2010). Of the two methods, capture by leg-hold snare was concluded to induce a greater stress response, based on higher serum concentrations of total cortisol (Cattet *et al.*, 2003; Chow *et al.*, 2010), as well as a greater probability of muscle injury, based on higher serum concentrations of myoglobin and the muscle enzymes aspartate aminotransferase and creatine kinase (Cattet *et al.*, 2008). These findings, however, contrast with the results of the present study in that HCC values were similar between bears captured by remote drug delivery from helicopter and bears captured by leg-hold snare. In addition, to meet the objectives for another study (M. Cattet*,* B.J. Macbeth, D.M. Janz and G.B. Stenhouse, unpublished observations), we evaluated the association between HCC and serum total cortisol concentration in 67 bears captured either by remote drug delivery from helicopter (*n* = 25) or by leg-hold snare (*n* = 42), and did not find any correlation (*r* = 0.012, *P* = 0.912).

In the present study, it was bears captured by culvert trap that tended to have the highest HCC values, a finding which could suggest that capture by culvert trap induced the strongest stress response of the three capture methods. Although we are not aware of any published reports that quantify the stress associated with capture by culvert trap, this again conflicts with (M. Cattet, B.J. Macbeth, D.M. Janz and G.B. Stenhouse, unpublished observations) on the haematology and serum biochemistry of brown bears, in which we have found that two markers of short-term or acute stress (i.e. occurring over minutes to hours), the neutrophil-to-lymphocyte ratio and serum total cortisol, show values in bears captured by culvert trap to be intermediate to the higher values in bears captured by leg-hold snare and to the lower values in bears captured by remote drug delivery from helicopter. We have also evaluated the association between HCC and serum total cortisol concentration in 18 bears captured by culvert trap, and again found no correlation (*r* = −0.176, *P* = 0.485). Thus, we are left with HCC results on the one hand that suggest an immediate response to capture, which varies in magnitude depending on the method of capture used, and with serum total cortisol values on the other hand that also vary quickly in response to different methods of capture, but in a distinctly different manner from the HCC.

The reason for the different response patterns between serum total cortisol and hair cortisol is not evident, but it does raise several questions. For example, is it because the central and peripheral stress axes respond differently to capture and that serum total cortisol is more influenced by the former while hair cortisol is more influenced by the latter? Is it because total cortisol reflects both protein-bound and unbound fractions (free cortisol) in the serum, whereas hair cortisol reflects only free cortisol? Or, is it because the HCC does not change in response to stress, but instead it is influenced by other unidentified factors that are unique to the method of capture? In this regard, Macbeth *et al.* (2010) reported previously on high HCC values in brown bears captured by culvert trap, and proposed that soiling of hair by urine, faeces and bait in the trap could alter the permeability of hair and, as a consequence, allow the influx of cortisol from these external contaminants. This proposed effect has since been substantiated in a controlled laboratory experiment, which demonstrated that cortisol from faeces and urine can increase the cortisol concentration of brown bear guard hair within 2 h of exposure, and that the amount of cortisol incorporated into the hair shaft is related to its concentration on the hair surface, as well as the duration of exposure (Macbeth, 2013). Although contamination may have occurred in some instances in our study, it is unlikely that it explains fully why the HCC values of bears captured by culvert trap were often greater than the HCC values of bears captured by the other two methods. Many bears captured by culvert trap were not visibly soiled and, even when soiled, field personnel preferentially sought dry, visibly clean hair samples. Another possibility is that the permeability of the hair of culvert trap-captured bears was also altered by increased humidity within the trap due to exhaled water vapour, and that this too facilitated the incorporation of external cortisol from sebum and sweat. However, if the HCC was increased by contamination and/or increased humidity in a significant and consistent manner, we would predict that the longer a bear was contained in a trap, the greater the increase in its HCC due to incorporation of external HCC. However, we did not find any association between HCC and restraint time for 17 bears captured by culvert trap.

Overall, a capacity for HCC to change rapidly, irrespective of external contamination, appears to offer the strongest explanation for our finding of differences in HCC between bears sampled following capture and bears snagged by barbed wire. However, further research is clearly needed to provide direct evidence of rapid change in HCC. A logical starting point would be to conduct adrenocorticotrophic hormone (ACTH) challenge tests on several captive brown bears to determine whether cortisol concentrations in hair are indicative of changes in the central stress axis as reflected by serum cortisol levels (Santymire *et al.*, 2012; Kersey and Dehnhard, 2014). We would suggest that ACTH is administered either as several injections or as a continuous infusion over a few hours to mimic better the minimal time (and presumably multiple stressors) typically required for the capture, handling and release of a brown bear. To date, ACTH challenge tests have been used to validate the measurement of hair cortisol in caribou and reindeer (both *Rangifer tarandus*; Ashley *et al.*, 2011) and in Canada lynx (*Lynx canadensis*; Terwissen *et al.*, 2013). Although HCC did not respond to a single ACTH challenge in caribou and reindeer, it did respond to weekly ACTH challenges over a 5 week period in Canada lynx. However, the challenge protocols used in both studies are quite different from what we are proposing in that they did not mimic the time frame and occurrence of multiple stressors that one would predict during the capture and handling of a wild mammal.

Hair cortisol concentration also appeared to be affected by capture and handling after hair growth had ceased and blood flow to follicles had terminated. Otherwise, we should not have detected differences in HCC between captured bears and barbed-wire-snagged bears, but we did. The median HCC for bears captured prior to mid-May and after mid-October, when hair growth had presumably ceased, was more than 2-fold greater than that of barbed-wire-snagged bears. Still, the timing of hair growth in brown bears shows a great deal of plasticity that depends on the quantity and quality of the diet (Jacoby *et al.*, 1999). In captive conditions, where high-quality food is plentiful, new hair growth and moulting of old hair begins in early May, with growth continuing at a relatively constant rate until October (personal communication from Dr Charles T. Robbins, Washington State University). However, in free-ranging conditions, nutrition may be limited during the spring and new growth delayed until early summer, when food availability increases (Hilderbrand *et al.*, 1996; Jacoby *et al.*, 1999; Hobson *et al.*, 2000). Although we cannot be certain that hair growth was arrested in all bears captured prior to mid-May and after mid-October, we generally do not observe bears moulting in Alberta before late May and we assume that hair growth has largely ended by mid-October, ∼3–4 weeks prior to hibernation. When hair is in a quiescent phase, its blood supply is essentially cut off (Stenn and Paus, 2001). Thus, if HCC increases in non-growing hair in response to capture, the cortisol must be integrated from sources other than blood. The peripheral stress axis within skin, including its epidermal and dermal compartments (e.g. sebaceous glands), as well as hair follicles, would seem to be likely sources, but rapid incorporation of cortisol into hair appears inconsistent with the structure of hair, which contains barriers to diffusion due to its low water and lipid content (Harkey, 1993; Meyer and Novak, 2012). The possibility of rapid diffusion is also inconsistent with the finding that cortisol concentration in brown bear hair (*n* = 15 samples) varies along its length and can differ more than 2-fold among proximal, middle and distal segments (unpublished work from Dr Bryan Macbeth, University of Calgary). Clearly, further research is required to confirm whether the cortisol concentration in non-growing hair is stable or, conversely, susceptible to rapid change. Again, the implementation of ACTH challenge tests in captive brown bears, when their hair growth is arrested, could help to resolve this dilemma.

The proportion of total variation in HCC explained by the fine-level analysis was less than that of the coarse-level analysis, i.e. 0.25 and 0.36, respectively. In addition, the predictor variables for the best models of the two analyses differed, with sex in the coarse-level model, a variable available to both analyses, replaced by age, BCI and Julian day in the fine-level model, which were variables available only to the latter analysis. Although mean age and mean Julian day were similar between females and males, the mean BCI for adult (≥5-year-old) males (1.19 ± 0.18, *n* = 38) was considerably greater than that for adult females (0.16 ± 0.11, *n* = 38), with differences being most pronounced during May–June, when the majority of captures occurred. Differences in body condition between subadult (<5-year-old) males (BCI = 0.47 ± 0.20, *n* = 15) and females (0.18 ± 0.35, *n* = 10) were less evident. Nevertheless, the BCI difference between adult males and females was enough to explain the apparent sex difference detected in the coarse-level analysis. Sex differences in the body condition of adult bears captured during spring have been noted previously for brown bears captured in this (Nielsen *et al.*, 2013) and other studies (e.g. McLellan, 2011).

Although our finding that HCC was inversely correlated with body condition in brown bears has not been reported previously, it has been reported for polar bears (*Ursus maritimus*; Macbeth *et al.*, 2012). Along the same lines, Bryan *et al.* (2013) reported that HCC for brown bears in coastal British Columbia, Canada, decreased with increasing dietary salmon availability, and was higher after a year of low than high salmon availability. Together, these findings suggest that HCC could be an indicator of nutritional stress, which is widely regarded as a form of long-term stress (Breuner *et al.*, 2013). However, the BCI may also serve as an indicator of other long-term stressors. For example, poor body condition can reflect the impact of abnormally increased energy consumption due to chronic disease (Tompkins *et al.*, 2011) or wound healing (Fuller *et al.*, 2011). Thus, high HHC combined with low BCI in a brown bear does not necessarily imply nutritional stress, and other information will be needed to clarify the specific stressor(s), e.g. intraspecific competition, human activities.

The median HCC of brown bears varied both annually and within years. Although activation of the central stress axis is likely to vary in frequency and/or intensity on an annual basis, as has recently been documented for brown bears with regard to nutritional stress (Bryan *et al.*, 2013), the yearly variation we documented was probably also confounded by year-to-year spatial differences in sampling. Hair samples were collected across five BMAs that differ significantly with respect to topography, habitat quality and human activity, but sampling per BMA was inconsistent over time. In 19 years of sampling from 1994 to 2012, all five BMAs were sampled in only 1 year (2008) and no more than two BMAs were sampled in 11 of the years. In fact, median HCC differed significantly (*P* ≤ 0.05) between some BMAs when year was removed from the coarse-level analysis, but when year and BMA were included together, BMA always came up as a non-significant factor.

Sampling by day within years was also inconsistent. Our findings suggest that HCC values tended to increase gradually and almost linearly from spring to autumn, but most of the data were weighted towards the months of May and June, with only 14 data points after 1 August. Nonetheless, the day-by-day pattern remained evident, even when we truncated the data to captures occurring prior to August. On the surface, a gradual increase in HCC throughout the year may seem counterintuitive, because body condition is improving over this time, and the association between HCC and body condition is inverse. However, HCC is a non-specific stress marker that may be influenced by a wide variety of stressors in addition to food availability, and perhaps the frequency and/or intensity of some of these stressors (e.g. encounters with humans) increases from spring to autumn. The fact that our best models explained no more than 36% of the total variation in HCC underscores the need for more research to identify other influential factors. On this note, a preliminary analysis of the FRIGBP data suggests that deviations in local weather (temperature and precipitation) and proximity to human activity may account for ∼30% of the total variation in HCC (S. Nielsen, J. Boulanger, M. Cattet, B.J. Macbeth, D.M. Janz and G.B. Stenhouse, unpublished observations).

### Conclusions

Our results support the view that HCC is influenced by long-term stress in brown bears, because HCC was inversely associated with body condition and was, therefore, presumably responsive to nutritional stress and/or possibly other long-term stressors that may affect body condition. However, our other findings suggest that HCC may also be influenced by short-term or acute stressors associated with capture, restraint and handling. In addition, our results suggest this effect may also occur when hair growth has terminated and blood supply to the hair follicle has essentially been cut off. The implications of these possible ‘acute effect’ findings are 2-fold. First, they challenge the common assumption that the primary determinant of HCC is passive diffusion from the vascular supply to the follicular cells that produce the hair. Clearly, further research is required to understand how the activities of the central and peripheral stress axes are linked and co-ordinated to affect HCC, and to what extent cortisol moves in and, possibly, out of hair as a consequence of changes in its permeability. Second, our results should caution researchers against using the HCC as an explicit indicator (biomarker) of long-term stress until further validation studies are completed. One of the most promising applications for the measurement of cortisol, and other hormones, in hair may be to enrich large-scale hair-trap studies by enabling the concurrent assessment of the health and fitness of wild, uncaptured animals. However, this first requires calibrating health and fitness measurements with hair hormone concentrations in live-captured and/or captive animals to identify threshold values that are needed to support conservation goals. If acute stressors can affect HCC rapidly at any time, as our results suggest, then we must identify and correct for these effects to ensure an accurate calibration, if at all possible. Conversely, measurement of cortisol in hair samples collected immediately following capture may provide a useful measure of short-term stress.

## Funding

This work was supported by Alberta Advanced Education and Technology (formerly Alberta Innovation and Science) [grant agreement IP-06-025A-SE]; Natural Sciences and Engineering Research Council of Canada [grant identification CRDPJ 328937–05]; partners of the Foothills Research Institute Grizzly Bear Program; Alberta Environment and Sustainable Resource Development; and the Morris Animal Foundation [grant number D08ZO–010].

## References

[1] Acevedo-Whitehouse K Duffus ALJ (2009) Effects of environmental change on wildlife health. Philos Trans R Soc B Biol Sci 364: 3429–3438. 10.1098/rstb.2009.0128 PMC2781848 19833653

[2] Alberta Sustainable Resource Development (SRD) (2010) Grizzly bears. Government of Alberta Sustainable Resource Development, http://esrd.alberta.ca/fish-wildlife/wildlife-management/bear-management/grizzly-bears/default.aspx

[3] Allenbach DM (2011) Fluctuating asymmetry and exogenous stress in fishes: a review. Rev Fish Biol Fisher 21: 355–376.

[4] Anderson DR (2008) Model Based Inference in the Life Sciences: A Primer on Evidence. Springer, New York, NY, pp 1–184.

[5] Anson JR Dickman CR Boonstra R Jessop TS (2013) Stress triangle: do introduced predators exert indirect costs on native predators and prey? PLoS ONE 8: e60916. 23585861 10.1371/journal.pone.0060916 PMC3621665

[6] Ashley NT Barboza PS Macbeth BJ Janz DM Cattet MRL Booth RK Wasser SK (2011) Glucocorticosteroid concentrations in feces and hair of captive caribou and reindeer following adrenocorticotropic hormone challenge. Gen Comp Endocrinol 172: 382–391. 21501613 10.1016/j.ygcen.2011.03.029

[7] Barrett ELB Burke TA Hammers M Komdeur J Richardson DS (2013) Telomere length and dynamics predict mortality in a wild longitudinal study. Mol Ecol 22: 249–259. 23167566 10.1111/mec.12110

[8] Bechshøft TØ Sonne C Rigét FF Letcher RJ Novak MA Henchey E Meyer JS Eulaers I Jaspers VLB Covaci A (2013) Polar bear stress hormone cortisol fluctuates with the North Atlantic Oscillation climate index. Polar Biol 36: 1525–1529.

[9] Boonstra R (2004) Coping with changing northern environments: the role of the stress axis in birds and mammals. Integr Comp Biol 44: 95–108. 21680490 10.1093/icb/44.2.95

[10] Bortolotti GR Marchant TA Blas J German T (2008) Corticosterone in feathers is a long-term, integrated measure of avian stress physiology. Funct Ecol 22: 494–500.

[11] Bortolotti GR Marchant T Blas J Cabezas S (2009) Tracking stress: localisation, deposition and stability of corticosterone in feathers. J Exp Biol 212: 1477–1482. 19411541 10.1242/jeb.022152

[12] Boulanger J Stenhouse G Proctor M Himmer S Paetkau D Cranston J (2005a) 2004 Population inventory and density estimates for the Alberta 3B and 4B brown bear management area. Report prepared for Alberta Sustainable Resource Development, Fish and Wildlife Division, May 2005 (with updates November 2005), Edmonton, Alberta, pp 1–28.

[13] Boulanger J Stenhouse G MacHutchon G Proctor M Himmer S Paetkau D Cranston J (2005b) Brown bear population and density estimates for the 2005 Alberta (proposed) unit 4 management area inventory. Report prepared for Alberta Sustainable Resource Development, Fish and Wildlife Division, December 2005, Edmonton, Alberta, pp 1–31.

[14] Boulanger J Stenhouse G MacHutchon G Proctor M Paetkau D Cranston J (2007) Brown bear population and density estimates for the 2006 Alberta unit 5 management area inventory. Report prepared for Alberta Sustainable Resource Development, Fish and Wildlife Division, May 2007, Alberta Sustainable Resource Development, Edmonton, Alberta, pp 1–37.

[15] Boulanger J MacHutchon G Stenhouse G Proctor M Cranston J Paetkau D (2008) Brown bear population and density estimates for Alberta Bear Management Unit 6 and British Columbia Management Units 4-1, 4-2, and 4-23 (2007). Report prepared for the Alberta Sustainable Resource Development, Fish and Wildlife Division, British Columbia Ministry of Forests and Range, British Columbia Ministry of Environment, and Parks Canada, pp 1–46.

[16] Breuner CW Delehanty B Boonstra R (2013) Evaluating stress in natural populations of vertebrates: total CORT is not good enough. Funct Ecol 27: 24–36.

[17] Bryan HM Darimont CT Paquet PC Wynne-Edwards KE Smits JEG (2013) Stress and reproductive hormones in brown bears reflect nutritional benefits and social consequences of a salmon foraging niche. PLoS ONE 8: e80537. 24312230 10.1371/journal.pone.0080537 PMC3842319

[18] Busch DS Hayward LS (2009) Stress in a conservation context: a discussion of glucocorticoid actions and how levels change with conservation-relevant variables. Biol Conserv 142: 2844–2853.

[19] Cattet MRL Caulkett NA Obbard ME Stenhouse GB (2002) A body condition index for ursids. Can J Zool 80: 1156–1161.

[20] Cattet MRL Christison K Caulkett NA Stenhouse GB (2003) Physiologic responses of brown bears to different methods of capture. J Wildl Dis 39: 649–654. 14567227 10.7589/0090-3558-39.3.649

[21] Cattet M Boulanger J Stenhouse G Powell RA Reynolds-Hogland MJ (2008) Long-term effects of capture and handling in ursids: implications for wildlife welfare and research. J Mammal 89: 973–990.

[22] Chow BA Hamilton J Alsop D Cattet MRL Stenhouse G Vijayan MM (2010) Brown bear corticosteroid binding globulin: cloning and serum protein expression. Gen Comp Endocrinol 167: 317–325. 20347821 10.1016/j.ygcen.2010.03.027

[23] Clinchy M Sheriff MJ Zanette LY (2013) Predator-induced stress and the ecology of fear. Funct Ecol 27: 56–65.

[24] Dantzer B Newman AEM Boonstra R Palme R Boutin S Humphries MM McAdam AG (2013) Density triggers maternal hormones that increase adaptive offspring growth in a wild mammal. Science 340: 1215–1217. 23599265 10.1126/science.1235765

[25] Fourie NH Bernstein RM (2011) Hair cortisol levels track phylogenetic and age related differences in hypothalamic–pituitary–adrenal (HPA) axis activity in non-human primates. Gen Comp Endocrinol 174: 150–155. 21893059 10.1016/j.ygcen.2011.08.013

[26] Fuller NW Reichard JD Nabhan ML Fellows SR Pepin LC Kunz TH (2011) Free-ranging little brown myotis (*Myotis lucifugus*) heal from wing damage associated with white-nose syndrome. EcoHealth 8: 154–162. 21922344 10.1007/s10393-011-0705-y

[27] Garshelis DL Gibeau ML Herrero S (2005) Brown bear demographics in and around Banff National Park and Kananaskis Country, Alberta. J Wildl Manage 69: 277–297.

[28] Harkey MR (1993) Anatomy and physiology of hair. Forensic Sci Int 63: 9–18. 8138238 10.1016/0379-0738(93)90255-9

[29] Harris EK (1988) Proposed goals for analytical precision and accuracy in single-point diagnostic testing. Arch Pathol Lab Med 112: 416–420. 3355343

[30] Herrero S, ed. (2005) Biology, demography, ecology and management of brown bears in and around Banff National Park and Kananaskis Country: the final report of the Eastern Slopes Grizzly Bear Project. Faculty of Environmental Design, University of Calgary, Calgary, Alberta, pp 1–248. http://www.canadianrockies.net/brown/final_report.html

[31] Hilderbrand GV Farley SD Robbins CT Hanley TA Titus K Servheen C (1996) Use of stable isotopes to determine diets of living and extinct bears. Can J Zool 74: 2080–2088.

[32] Hobson KA McLellan BN Woods JG (2000) Using stable carbon (δ^13^C) and nitrogen (δ^15^N) isotopes to infer trophic relationships among black and brown bears in the upper Columbia River basin, British Columbia. Can J Zool 78: 1332–1339.

[33] Hodges KE Boonstra R Krebs CJ (2006) Overwinter mass loss of snowshoe hares in the Yukon: starvation, stress, adaptation or artefact? J Anim Ecol 75: 1–13. 16903038 10.1111/j.1365-2656.2005.01018.x

[34] Ito N Ito T Kromminga A Bettermann A Takigawa M Kees F Straub RH Paus R (2005) Human hair follicles display a functional equivalent of the hypothalamic-pituitary-adrenal axis and synthesize cortisol. FASEB J 19: 1332–1334. 15946990 10.1096/fj.04-1968fje

[35] Jachowski DS Slotow R Millspaugh JJ (2012) Physiological stress and refuge behavior by African elephants. PLoS ONE 7: e31818. 22384079 10.1371/journal.pone.0031818 PMC3284500

[36] Jacoby ME Hilderbrand GV Servheen C Schwartz CC Arthur SM Hanley TA Robbins CT Michener R (1999) Trophic relations of brown and black bears in several western North American ecosystems. J Wildl Manage 63: 921–929.

[37] Johnstone CP Reina RD Lill A (2012) Interpreting indices of physiological stress in free-living vertebrates. J Comp Physiol B 182: 861–879. 22415475 10.1007/s00360-012-0656-9

[38] Keckeis K Lepschy M Schöpper H Moser L Troxler J Palme R (2012) Hair cortisol: a parameter of chronic stress? Insights from a radiometabolism study in guinea pigs. J Comp Physiol B 182: 985–996. 22592890 10.1007/s00360-012-0674-7

[39] Kersey DC Dehnhard M (2014) The use of noninvasive and minimally invasive methods in endocrinology for threatened mammalian species conservation. Gen Comp Endocrinol http://dx.doi.org/10.1016/j.ygcen.2014.04.022. 10.1016/j.ygcen.2014.04.022 24798579

[40] Koren L Mokady O Karaskov T Klein J Koren G Geffen E (2002) A novel method using hair for determining hormonal levels in wildlife. Anim Behav 63: 403–406.

[41] Koren L Mokady O Geffen E (2008) Social status and cortisol levels in singing rock hyraxes. Horm Behav 54: 212–216. 18423638 10.1016/j.yhbeh.2008.02.020

[42] Koren L Nakagawa S Burke T Soma KK Wynne-Edwards KE Geffen E (2012) Non-breeding feather concentrations of testosterone, corticosterone and cortisol are associated with subsequent survival in wild house sparrows. Proc Biol Sci 279: 1560–1566. 22090380 10.1098/rspb.2011.2062 PMC3282351

[43] Lendvai ÁZ Giraudeau M Németh J Bakó V McGraw KJ (2013) Carotenoid-based plumage coloration reflects feather corticosterone levels in male house finches (*Haemorhous mexicanus*). Behav Ecol Sociobiol 67: 1817–1824.

[44] Macbeth BJ (2013) An evaluation of hair cortisol concentration as a potential biomarker of long-term stress in free-ranging brown bears (*Ursus arctos*), polar bears (*Ursus maritimus*), and caribou (*Rangifer tarandus* sp.). PhD thesis University of Saskatchewan, Saskatoon, Saskatchewan.

[45] Macbeth BJ Cattet M Stenhouse GB Gibeau ML Janz DM (2010) Hair cortisol concentration as a non-invasive measure of long-term stress in free-ranging brown bears (*Ursus arctos*): considerations with implications for other wildlife. Can J Zool 88: 935–949.

[46] Macbeth BJ Cattet M Obbard ME Middel K Janz DM (2012) Evaluation of hair cortisol concentration as a biomarker of long-term stress in free-ranging polar bears. Wildl Soc Bull 36: 747–758.

[47] McLellan BN (2011) Implications of a high-energy and low-protein diet on the body composition, fitness, and competitive abilities of black (*Ursus americanus*) and brown (*Ursus arctos*) bears. Can J Zool 89: 546–558.

[48] Meyer JS Novak MA (2012) Minireview: Hair cortisol: a novel biomarker of hypothalamic-pituitary-adrenocortical activity. Endocrinology 153: 4120–4127. 22778226 10.1210/en.2012-1226 PMC3423616

[49] Miño CI Del Lama SN (2009) Molted feathers as a source of DNA for genetic studies in waterbird populations. Waterbirds 32: 322–329.

[50] Mizutani Y Tomita N Niizuma Y Yoda K (2013) Environmental perturbations influence telomere dynamics in long-lived birds in their natural habitat. Biol Lett 9: 20130511 http://dx.doi.org/10.1098/rsbl.2013.0511 23945210 10.1098/rsbl.2013.0511 PMC3971690

[51] Monaghan P Metcalfe NB Torres R (2009) Oxidative stress as a mediator of life history trade-offs: mechanisms, measurements and interpretation. Ecol Lett 12: 75–92. 19016828 10.1111/j.1461-0248.2008.01258.x

[52] Nielsen SE Cattet MRL Boulanger J Cranston J McDermid GJ Shafer ABA Stenhouse GB (2013) Environmental, biological and anthropogenic effects on brown bear body size: temporal and spatial considerations. BMC Ecol 13: 31. 24010501 10.1186/1472-6785-13-31 PMC3849066

[53] Paetkau D (2003) Genetical error in DNA-based inventories: insight from reference data and recent projects. Mol Ecol 12: 1375–1387. 12755868 10.1046/j.1365-294x.2003.01820.x

[54] Proctor M McLellan B Boulanger J Apps C Stenhouse G Paetkau D Mowat G (2010) Ecological investigations of brown bears in Canada using DNA from hair, 1995–2005: a review of methods and progress. Ursus 21: 169–188.

[55] R Development Core Team (2013) R: A Language and Environment for Statistical Computing. R Foundation for Statistical Computing, Vienna, Austria ISBN 3-900051-07-0, http://www.R-project.org/.

[56] Rodrigues da Paz RC Souza NP Brown JL (2014) Evaluation of glucocorticoid faecal monitoring as a non-invasive assessment of stress in captive crab-eating fox (*Cerdocyoun thous*) after ACTH stimulation. J Steroids Hormon Sci S12: 008 doi:10.4172/2157-7536.S12-008.

[57] Russell E Koren G Rieder M Van Uum S (2012) Hair cortisol as a biological marker of chronic stress: current status, future directions and unanswered questions. Psychoneuroendocrinology 37: 589–601. 21974976 10.1016/j.psyneuen.2011.09.009

[58] Russell E Koren G Rieder M Van Uum S (2014) The detection of cortisol in human sweat: implications for measurement of cortisol in hair. Ther Drug Monit 36: 30–34. 24216536 10.1097/FTD.0b013e31829daa0a

[59] Sánchez-Chardi A García-Pando M López-Fuster MJ (2013) Chronic exposure to environmental stressors induces fluctuating asymmetry in shrews inhabiting protected Mediterranean sites. Chemosphere 93: 916–923. 23800592 10.1016/j.chemosphere.2013.05.056

[60] Santymire RM Freeman EW Lonsdorf EV Heintz MR Armstrong DM (2012) Using ACTH challenges to validate techniques for adrenocortical activity analysis in various African wildlife species. Int J Anim Vet Adv 4: 99–108.

[61] Schielzeth H (2010) Simple means to improve the interpretability of regression coefficients. Methods Ecol Evol 1: 103–113.

[62] Schultner J Kitaysky AS Gabrielsen GW Hatch SA Bech C (2013) Differential reproductive responses to stress reveal the role of life-history strategies within a species. Proc Biol Sci 280: 20132090 http://dx.doi.org/10.1098/rspb.2013.2090 24089339 10.1098/rspb.2013.2090 PMC3790493

[63] Selman C Blount JD Nussey DH Speakman JR (2012) Oxidative damage, ageing, and life-history evolution: where now? Trends Ecol Evol 27: 570–577. 22789512 10.1016/j.tree.2012.06.006

[64] Sharpley CF Kauter KG McFarlane JR (2010a) An investigation of hair cortisol concentration across body sites and within hair shaft. Clin Med Insights Endocrinol Diabetes 3: 17–23. 22879783 10.4137/cmed.s4465 PMC3411542

[65] Sharpley CF Kauter KG McFarlane JR (2010b) Hair cortisol concentration differs across site and person: localisation and consistency of responses to a brief pain stressor. Physiol Res 59: 979–983. 20533859 10.33549/physiolres.931874

[66] Sharpley CF McFarlane JR Slominski A (2011) Stress-linked cortisol concentrations in hair: what we know and what we need to know. Rev Neurosci 23: 111–121. 22150070 10.1515/RNS.2011.058 PMC3381079

[67] Sheriff MJ Love OP (2013) Determining the adaptive potential of maternal stress. Ecol Lett 16: 271–280. 23205937 10.1111/ele.12042

[68] Sheriff MJ Dantzer B Delehanty B Palme R Boonstra R (2011a) Measuring stress in wildlife: techniques for quantifying glucocorticoids. Oecologia 166: 869–887. 21344254 10.1007/s00442-011-1943-y

[69] Sheriff MJ Krebs CJ Boonstra R (2011b) From process to pattern: how fluctuating predation risk impacts the stress axis of snowshoe hares during the 10-year cycle. Oecologia 166: 593–605. 21246218 10.1007/s00442-011-1907-2

[70] Slominski A Mihm MC (1996) Potential mechanism of skin response to stress. Int J Dermatol 35: 849–851. 8970839 10.1111/j.1365-4362.1996.tb05049.x

[71] Slominski A Wortsman J Tuckey RC Paus R (2007) Differential expression of HPA axis homolog in the skin. Mol Cell Endocrinol 265–266: 143–149. 10.1016/j.mce.2006.12.012 PMC1839836 17197073

[72] Stalder T Kirschbaum C (2012) Analysis of cortisol in hair – State of the art and future directions. Brain Behav Immun 26: 1019–1029. 22366690 10.1016/j.bbi.2012.02.002

[73] Stenhouse GB Graham K, eds. (2011) Foothills Research Institute Grizzly Bear Program 2010 annual report. Foothills Research Institute, Hinton, Alberta, pp 1–180.

[74] Stenn KS Paus R (2001) Controls of hair follicle cycling. Physiol Rev 81: 449–494. 11152763 10.1152/physrev.2001.81.1.449

[75] Strasser EH Heath JA (2013) Reproductive failure of a human-tolerant species, the American kestrel, is associated with stress and human disturbance. J Appl Ecol 50: 912–919.

[76] Terwissen CV Mastromonaco GF Murray DL (2013) Influence of adrenocorticotrophin hormone challenge and external factors (age, sex, and body region) on hair cortisol concentration in Canada lynx (*Lynx canadensis*). Gen Comp Endocrinol 194: 162–167. 24080086 10.1016/j.ygcen.2013.09.010

[77] Thomson S Koren G Fraser LA Rieder M Friedman TC Van Uum SHM (2009) Hair analysis provides a historical record of cortisol levels in Cushing's syndrome. Exp Clin Endocrinol Diabetes 118: 133–138. 19609841 10.1055/s-0029-1220771 PMC2945912

[78] Tompkins DM Dunn AM Smith MJ Telfer S (2011) Wildlife diseases: from individuals to ecosystems. J Anim Ecol 80: 19–38. 20735792 10.1111/j.1365-2656.2010.01742.x

[79] von der Ohe CG Wasser SK Hunt KE Servheen C (2004) Factors associated with fecal glucocorticoids in Alaskan brown bears (*Ursus arctos horribilis*). Physiol Biochem Zool 77: 313–320. 15095251 10.1086/378139

[80] Wasser SK Hunt KE Brown JL Cooper K Crockett CM Bechert U Millspaugh JJ Larson S Monfort SL (2000) A generalized fecal glucocorticoid assay for use in a diverse array of nondomestic mammalian and avian species. Gen Comp Endocrinol 120: 260–275. 11121291 10.1006/gcen.2000.7557

[81] Wasser SK Keim JL Taper ML Lele SR (2011) The influences of wolf predation, habitat loss, and human activity on caribou and moose in the Alberta oil sands. Front Ecol Environ 9: 546–551.

[82] Wingfield JC (2013) Ecological processes and the ecology of stress: the impacts of abiotic environmental factors. Funct Ecol 27: 37–44.

[83] Wingfield JC Kelley JP Angelier F Chastel O Lei F Lynn SE Miner B Davis JE Li D Wang G (2011) Organism–environment interactions in a changing world: a mechanistic approach. J Ornithol 152 Suppl 1: S279–S288.

[84] Wood SN (2006) Generalized Additive Models: an Introduction with R. Chapman and Hall, CRC Texts in Statistical Science, London, UK, pp 1–392.

[85] Wood SN (2013) Mixed GAM computation vehicle with GCV/AIC/REML smoothness estimation. CRAN Version 1.7-23, pp 1–215.

[86] Woods JG McLellan BN Paetkau D Proctor M Lewis D Strobeck C (1999) Genetic tagging free-ranging black and brown bears. Wildl Soc Bull 27: 616–627.

[87] Zmijewski MA Slominski AT (2011) Neuroendocrinology of the skin – An overview and selective analysis. Dermato-endocrinology 3: 3–10. 21519402 10.4161/derm.3.1.14617 PMC3051846

[88] Zuur AF (2012) Beginner's Guide to Generalized Additive Models with R. Highland Statistics Ltd, Newburgh, UK, pp 1–206.

[89] Zuur AF Ieno EN Walker NJ Saveliev AA Smith GM (2009) Mixed Effects Models and Extensions in Ecology with R. Springer, New York, NY, pp 1–574.

[90] Zuur AF Saveliev AA Ieno EN (2014) A Beginner's Guide to Generalized Additive Mixed Models with R. Highland Statistics Ltd, Newburgh, UK, pp 1–332.

[91] Zwijacz-Kozica T Selva N Barja I Silván G Martínez-Fernández L Illera JC Jodłowski M (2013) Concentration of fecal cortisol metabolites in chamois in relation to tourist pressure in Tatra National Park (South Poland). Acta Theriol 58: 215–222.

